# RNAi-mediated knockdown of *Parp1* does not improve the development of female cloned mouse embryos

**DOI:** 10.18632/oncotarget.19418

**Published:** 2017-07-18

**Authors:** Guang-Yu Bai, Si-Hang Song, Rui-Zhen Sun, Zi-Hui Zhang, Jingyu Li, Zhen-Dong Wang, Zhong-Hua Liu, Lei Lei

**Affiliations:** ^1^ Department of Histology and Embryology, Harbin Medical University, Harbin, 150081, China; ^2^ Laboratory of Embryo Biotechnology, College of Life Science, Northeast Agricultural University, Harbin, 150030, China

**Keywords:** PARP1, X chromosome, rDNA, nuclear transfer

## Abstract

Somatic cell nuclear transfer is an important technique for life science research, but its efficiency is still extremely low, and most genes that are important during early development, such as X chromosome-linked genes, are not appropriately expressed during this process. Poly (ADP-ribose) polymerase (PARP) is an enzyme that transfers ADP ribose clusters to target proteins. PARP family members such as PARP1 participate in cellular signalling pathways through poly (ADP-ribosylation) (PARylation), which ultimately promotes changes in chromatin structure, gene expression, and the localization and activity of proteins that mediate signalling responses. PARP1 is associated with X chromosome inactivation (Xi). Here, we showed that abnormal Xi occurs in somatic cell nuclear transfer (NT) blastocysts, whereas in female blastocysts derived from cumulus cell nuclear transfer, both X chromosomes were inactive. *Parp1* expression was higher in female NT blastocysts than that in intracytoplasmic sperm injection (ICSI) embryos but not in male NT blastocysts. After knocking down *Parp1* expression, both the pre-rRNA 47S and X-inactivation-specific transcript (*Xist*) levels increased. Moreover, the expression of genes on the inactivated X chromosome, such as *Magea6* and *Msn,* were also increased in the NT embryos. However, the development of Parp1si NT embryos was impaired, although total RNA sequencing showed that overall gene expression between the Parp1si NT blastocysts and the control was similar. Our findings demonstrate that increases in the expression of several genes on the X chromosome and of rRNA primary products in NT blastocysts with disrupted *Parp1* expression are insufficient to rescue the impaired development of female cloned mouse embryos and could even exacerbate the associated developmental deficiencies.

## INTRODUCTION

Somatic cell nuclear transfer is an important technique for life science research that has been widely applied in animal husbandry and breeding, in the protection of endangered animal species, and in medical research on stem cells and organs for tissue reconstruction. Despite the widespread use of this technique, however, its efficiency is still very low. Many studies have revealed large differences in gene expression between nuclear transfer (NT) embryos and normal fertilized embryos [1–4], especially during early stages of development and for X chromosome-linked genes. *Inoue* et al. revealed significant down-regulation of genes on the X chromosome in cloned embryos and demonstrated that X-inactivation-specific transcript (*Xist*) was ectopically expressed on the active X chromosome in female and male cloned embryos. Consequently, the single X chromosome in male cloned embryos and both X chromosomes in female cloned embryos were abnormally inactivated. Subsequently, Ogura’s group achieved considerable development of cloned embryos through knocking out or knocking down *Xist* in donor cells [5, 6].

It has been suggested that poly(ADP-ribose) polymerases (PARPs) are key factors in the formation of facultative heterochromatin in the inactive X chromosome [7]. PARP family members transfer ADP ribose clusters to target proteins and thus interfere with their activity in the nucleus [8]. PARP1 participates in cellular signalling pathways through poly (ADP-ribosylation) (PARylation). This process alters chromatin architecture, gene expression, and the localization and activity of proteins that mediate signalling responses [9]. PARP1 participates in the establishment and maintenance of rDNA heterochromatin and is associated with inactivation of the X chromosome [7, 10].

In a previous study, we demonstrated that the efficiency of ribosomal DNA (rDNA) reprogramming in NT embryos is determined by rDNA activity in the donor cells from which they are derived. DNA methylation of rDNA promoters is not fully reprogrammed in oocytes [11]. Heterochromatin must be remodelled during the reprogramming of somatic cells in NT embryos. As a component of the silencing complex, PARP1 should be involved in this process. Given that rDNA demethylation is incomplete and that Xi is abnormal in NT embryos, we hypothesized that knocking down *Parp1* might promote the development of NT embryos by alleviating rDNA heterochromatin or Xi.

The objective of the present study was to investigate the influence of PARP1 on rDNA transcription and X-linked gene expression during the early development of mouse NT embryos.

## RESULTS

### Both X chromosomes in CCNT embryos are inactive, and *Parp1* levels are increased in female NT blastocysts

**Figure 1:**
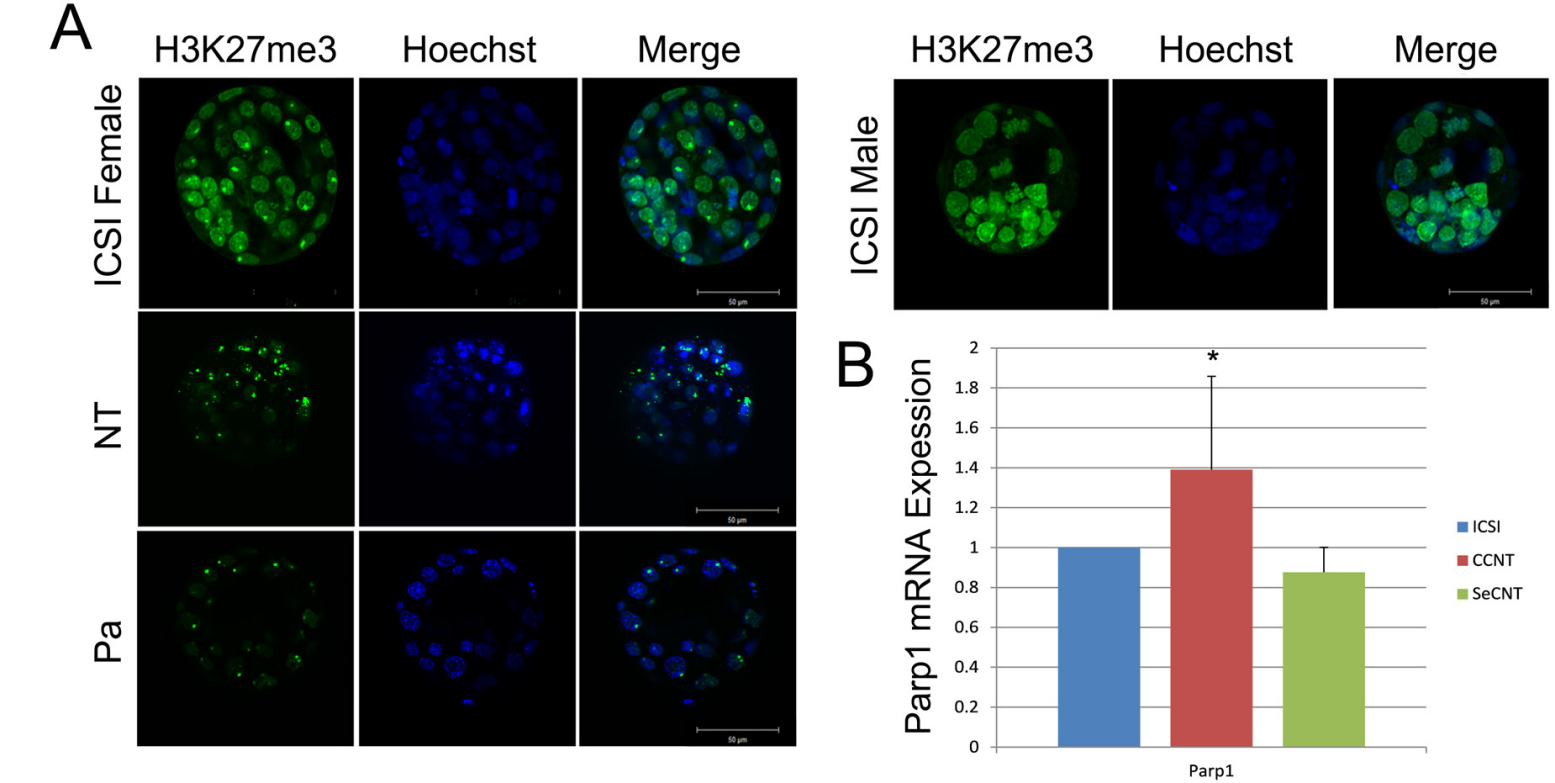
Distribution of H3K27me3 in ICSI and CCNT blastocysts and Real-time quantitative expression of *Parp1* **(A)** Distribution of H3K27me3 in ICSI female and male, CCNT, and parthenogenetic blastocysts. ICSI: intracytoplasmic sperm injection, CCNT: CCs as the donor for nuclear transfer, P: parthenogenesis. H3K27me3 is presented in green, whereas DNA is stained by Hoechst in blue. The merge shows both H3K27me3 and DNA. **(B)** *Parp1* expression level in CCNT, SeCNT and ICSI embryos at the blastocyst stage. SeCNT: Sertoli cells as the donor for nuclear transfer. The expression level in ICSI embryos was set as 1. * Indicates significant differences at P <0.05.

In mammals, the difference in the chromosome complement between males and females is achieved through the silencing of the genes on one of the two X chromosomes in females. Thus, in both male and female cells, only a single copy of the X chromosome is active [12]. The *Xist* gene is exclusively expressed from the inactive X chromosome and has been suggested to act as a non-coding RNA based on the convincing argument that the majority of *Xist* RNA localizes to the nucleus and, more specifically, accumulates within the territory of the inactive chromosome. The Ogura group confirmed that the localization of trimethylated histone H3 at lysine 27 (H3K27me3) is responsible for the repressive chromatin state in the inactive X chromosome [5, 6]. Thus, we used H3K27me3 as a marker of Xi and identified one inactive X chromosome per blastomere in female ICSI embryos or parthenogenetic embryos and no inactive X chromosomes in males (Figure 1A). These results confirmed that H3K27me3 is an effective marker of Xi. Then, we detected the fluorescent signal of H3K27me3 in CCNT embryos and found two inactive X chromosomes in each blastomere of CCNT blastocysts (Figure 1A). These observations suggest that abnormal Xi occurred in CCNT embryos, possibly explaining their impaired development.

PARP1 plays an important role in Xi reactivation [7]. To measure gender differences in the expression of *Parp1*, we constructed NT embryos of both sexes using CCs and Sertoli cells as donors. Through Q-PCR, we found that *Parp1* levels were significantly increased in female NT blastocysts compared with ICSI embryos (Figure 1B), but this increase was not observed in male NT blastocysts. Therefore, we decided to reduce the expression of *Parp1* in female NT embryos.

### Knocking down *Parp1* partially reactivates Xi

PARPs are enzymes that transfer ADP-ribose groups to target proteins. PARPs act through a complex response network that is driven by the cellular, molecular and chemical biology of poly(ADP-ribose) (PAR). A role for PARP1 in Xi has been suggested based on the protein’s association with the C-terminal non-histone domain of the histone variant MACROH2A1.2, a key factor in the stable silencing of genes on the inactive X chromosome [13]. Given the increased expression of *Parp1* that was observed in NT blastocysts, we decided to reduce *Parp1* levels to reactivate the inactivated X chromosome.

**Figure 2:**
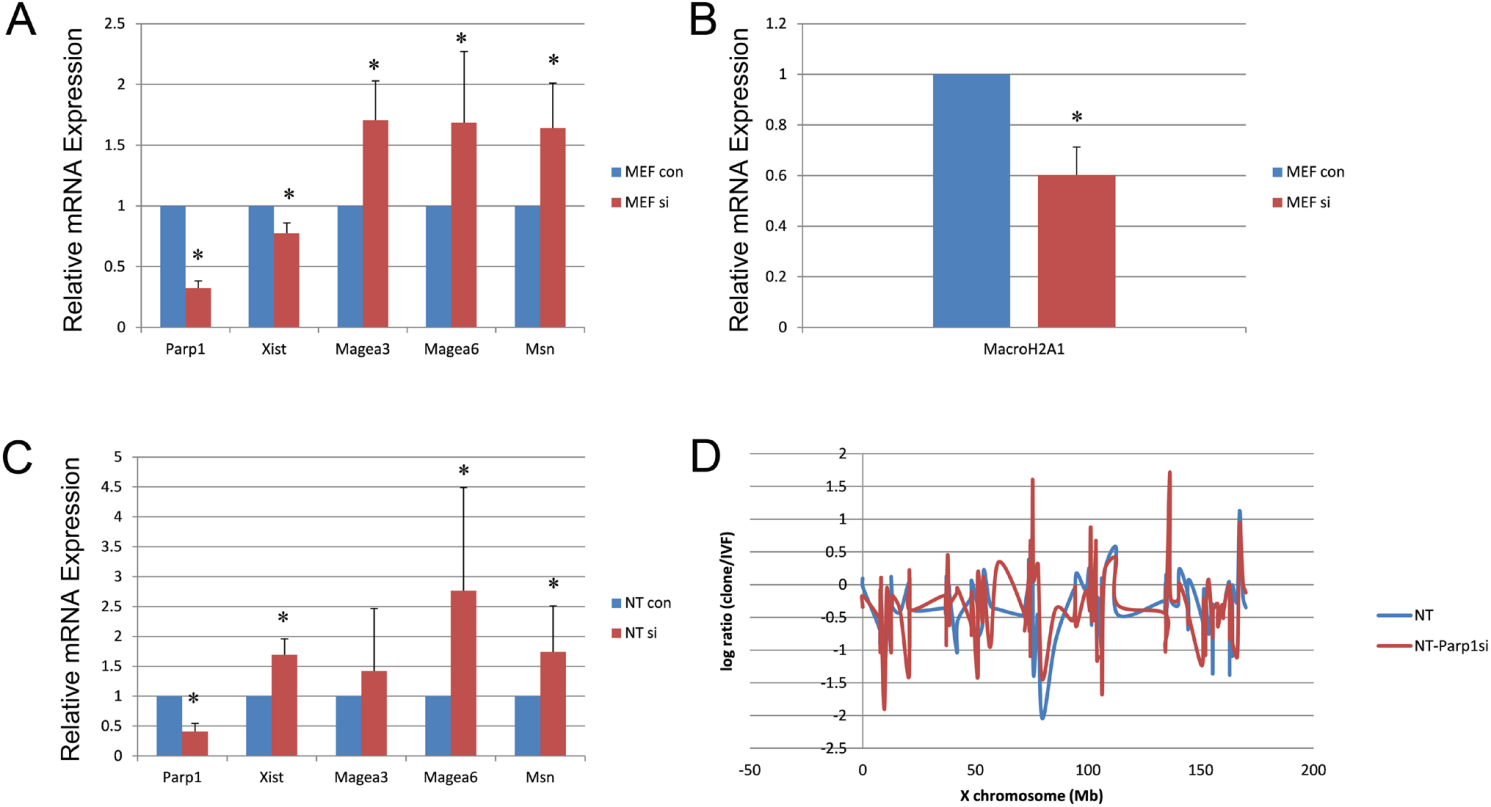
Expression of X chromosome-linked genes in MEF and NT blastocysts when *Parp1* was knocked down **(A)** Expression of some X chromosome-related genes in MEFs when *Parp1* was knocked down. **(B)** Expression of MacroH2A1 in MEFs when *Parp1* was knocked down. **(C)** Expression of X chromosome-related genes in NT blastocysts when *Parp1* was knocked down. The expression level of the si control was set as 1. * indicates significant differences at P <0.05 **(D)** The relative expression levels of all X-linked genes plotted at the X chromosome position in cloned embryos.

Using *Parp1* siRNA at a concentration of 100 nM with a reaction time of 72 h, we reduced the *Parp1* expression level to 20% in MEFs (Supplementary Figure 1). Immunofluorescence showed that PARP1 was rapidly reduced in NT blastocysts following *Parp1* siRNA treatment (Supplementary Figure 2). After knocking down the expression of *Parp1*, *Xist* levels decreased in female MEFs, whereas the expression of genes on the X chromosome, such as *Magea3*, *Magea6* and *Msn,* increased (Figure 2A). The expression of the histone variant *MacroH2A1*, a key factor in the stable silencing of genes on the inactive X chromosome, decreased after *Parp1* expression was reduced (Figure 2B). In NT blastocysts, the X chromosome genes *Magea6* and *Msn* exhibited increased expression when *Parp1* expression was reduced (Figure 2C). We also detected the expression of all X chromosome-linked genes in NT blastocysts and identified various genes that showed increases when *Parp1* expression was reduced (Figure 2D). These results suggest that knocking down *Parp1* partially reduced Xi. However, *Xist* expression also increased in blastocysts in which *Parp1* expression was reduced. This phenomenon was different from that observed in MEFs, indicating that PARP1 might participate in Xi through other signalling pathways in CCNT cloned embryos.

### Knocking down *Parp1* impairs NT embryo development

**Figure 3:**
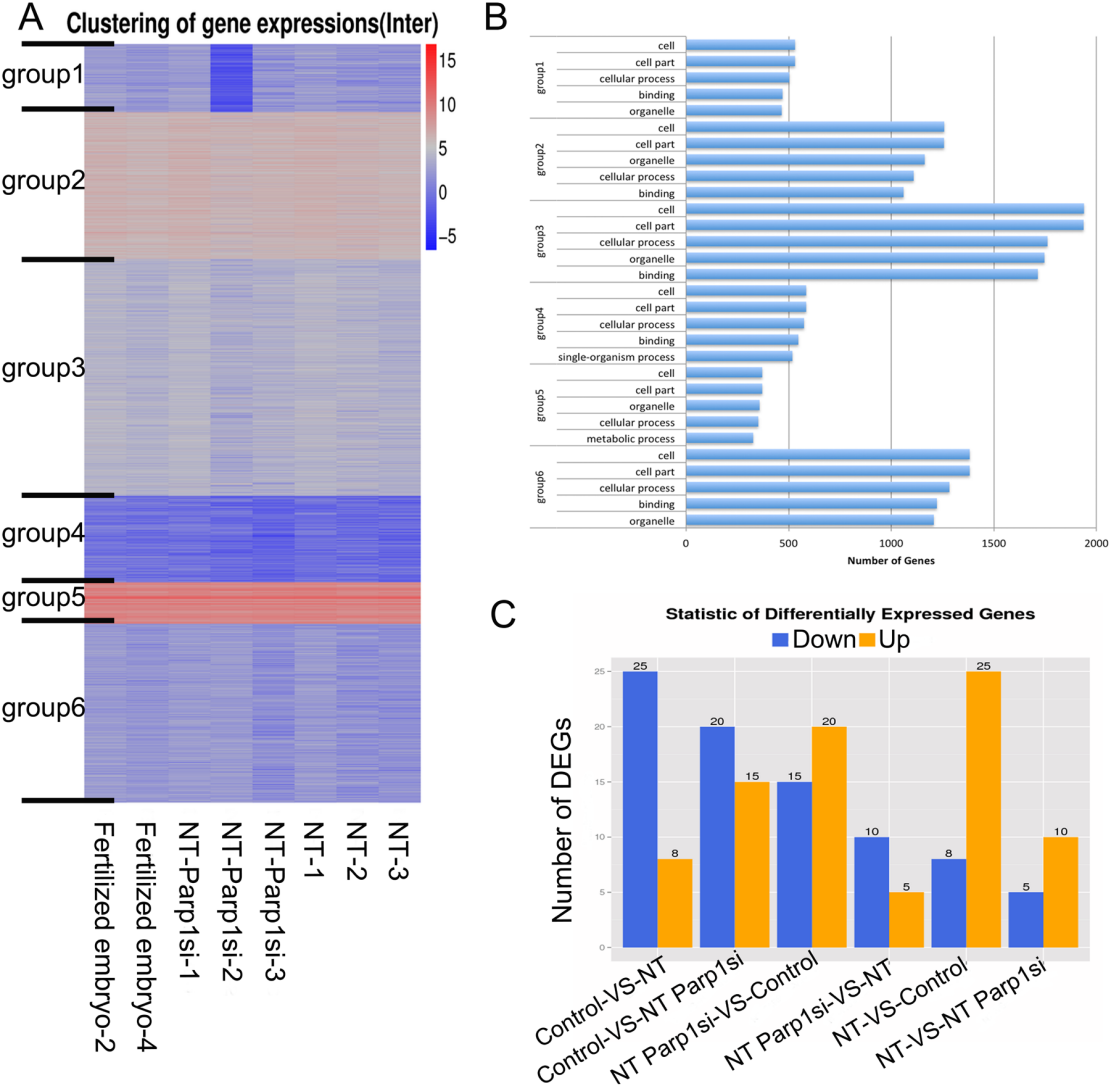
RNA-Seq was performed, and no differences were observed among the NT, NT-Parp1si and Female Fertilized blastocysts **(A)** Heatmap illustrating overall gene expression in the three types of blastocysts. **(B)** Gene ontology analysis of the six groups classified in A. The total genes were classified into six groups via unsupervised hierarchical clustering. **(C)** Differentially expressed genes (DEGs) between 2 of the 3 types of blastocysts.

Although the expression of some X chromosome-linked genes increased after *Parp1* expression was reduced, corresponding increases in the blastocyst rate of the NT embryos were not observed, given that the rate was reduced by approximately 2-fold in the *Parp1*si-NT embryos (18.5%) compared with the control NT embryos (43.2%), and none of the *Parp1*si-NT offspring was got after embryo transfer compared with control (Table 1). We hypothesized that PARP1 plays an important role in the development of NT embryos and to address this hypothesis, we performed expression profiling of the blastocysts to be sequenced. The total RNA sequence showed minimal differences in gene expression between the *Parp1*si NT blastocysts and the female controls (Figure 3A), and fewer than 30 DEGs were identified among the Fertilized, NT, and NT-Parp1si embryos (Figure 3C). All the expressed genes could be classified into six groups through unsupervised hierarchical cluster analysis (Figure 3A). GO analysis revealed that these genes were similar (Figure 3B), indicating that once the embryos developed to the blastocyst stage, gene expression in NT and NT-Parp1si embryos was similar to that in Fertilized embryos. However, NT embryo development was impaired when *Parp1* expression was reduced, possibly due to the other important roles of PARP1, such as maintenance of DNA integrity and DNA repair [14, 15], which were altered during early development. Osada, Nozaki [14] found that DNA damage can occur in the nuclear reprogramming process during cloning. As a component of DNA repair, PARP1 is likely involved in this process. Thus, the development of NT embryos is impaired when *Parp1* is knocked down.

**Table 1:** Rate of development of nuclear transfer embryos after *Parp1* interference

Groups	Embryos	2-cell (%)	4-cell (%)	Morula (%)	Blastocysts (%)	Offspring (%)
Control	137	125 (91.41±4.87)^a^	91 (73.11±10.27)^a^	72 (57.84±12.12)^a^	54 (43.37±4.47)^a^	1 (0.78±1.5)
*Parp1* siRNA	140	130 (92.31±2.62)^a^	111 (82.44±7.84)^a^	55 (43.32±3.20)^a^	24 (18.05±3.82)^b^	0 (0)

The experiment was repeated three times.

^a^ Values with different superscripted symbols in the same column differ significantly (P<0.05).

^b^ Values with different superscripted symbols in the same column differ significantly (P<0.05).

### Knocking down *Parp1* increases rRNA transcription in NT embryos

**Figure 4:**
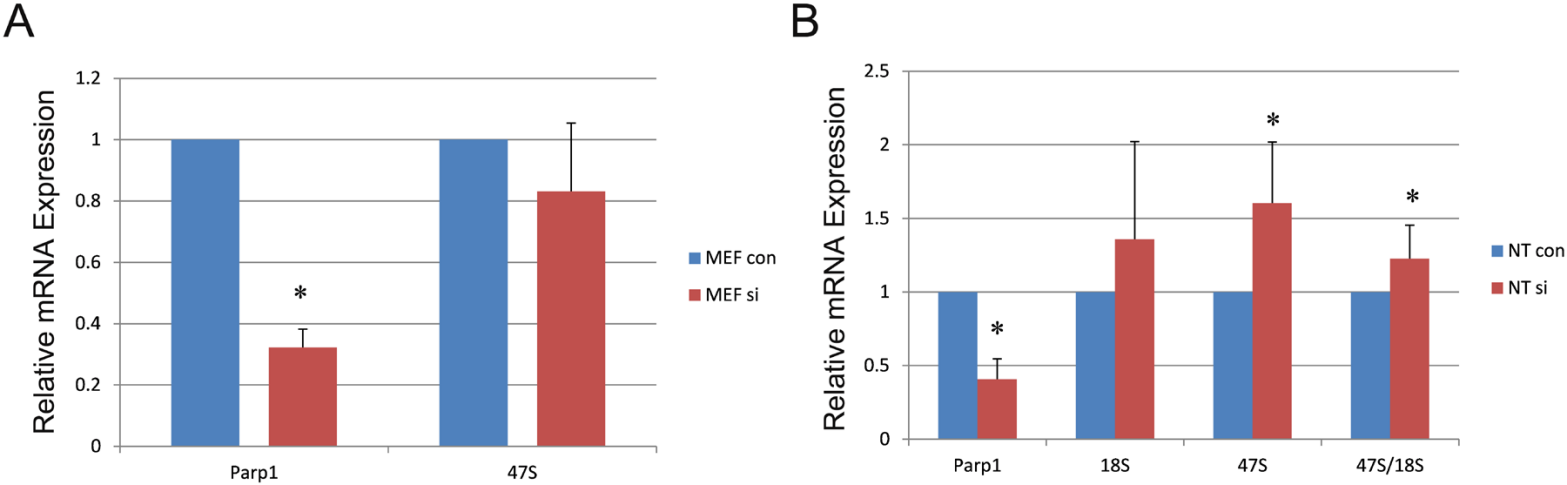
Effects of rDNA-related gene expression in MEF and NT blastocysts when *Parp1* was knocked down **(A)** Expression of 47S in MEFs when *Parp1* was knocked down. **(B)** Expression of 47S and 18S in NT blastocysts when *Parp1* was knocked down. The expression level of the si control was set as 1. * indicates significant differences at P <0.05

PARP1 participates in the establishment and maintenance of rDNA heterochromatin [16, 17]. rDNA activity is essential for NT development. Therefore, we detected the head product and end product of rDNA (47S and 18S) in female MEF and NT blastocysts. The expression of 47S did not change in female MEFs when *Parp1* expression was reduced; however, its expression increased in NT blastocysts after *Parp1* was knocked down. In contrast, 18S levels showed no changes in NT blastocysts (Figure 4).

## DISCUSSION

### Knocking down Parp1 only partially reactivates Xi and does not rescue the impaired development of female cloned embryos

In this study, we confirmed that both X chromosomes are inactive in NT embryos. Although reducing *Parp1* expression partially reactivated the X chromosome, the development of NT embryos was not significantly improved.

We hypothesize that the following explanations might account for these findings. First, PARP1 not only participates in maintaining Xi but also plays important roles in other processes, such as rDNA heterochromatinization [15, 16, 18]. Zhang, Huynh [19] reported that the inactive X chromosome is located near a distinct nuclear compartment after it pairs with its homologous partner in female MEFs. During mid-to-late S-phase, approximately 80 to 90% of inactivated X chromosomes contact the nucleolus. When ectopic *Xist* is knocked-in in autosomes, they also localize near the perinucleolar compartment. Deleting *Xist* results in a loss of nucleolar association [19]. rDNA is located in the perinucleolar compartment; thus, the inactivated X chromosome colocalizes with rDNA heterochromatin. Recent results identified interactions between PARP1 and TTF-I-interacting protein 5 (TIP5), which is part of the nucleolar remodelling complex (NoRC). The NoRC controls the maintenance of silent rDNA chromatin. This connection is mediated by pRNA and is implemented to accumulate PARP1 at rRNA genes to rebuild silent chromatin after replication in mid-late S-phase [20, 21]. Knockdown and overexpression of PARP1 not only interfere with rDNA heterochromatin but also influence the chromatin structure of human α-satellite repeats [22, 23]. We found that *Parp1*si activated rDNA transcription in NT blastocysts. However, the expression of the end product 18S was not increased in either MEF or NT embryos after *Parp1*si.

It had been reported that depletion of PARP-1 by RNA interference caused reactivation of a reporter gene on the inactive X chromosome, demonstrating that PARP-1 participated in the maintenance of silencing [7]. PARP1 also operate at heterochromatic regions such as telomeres, pericentric heterochromatin and silent ribosomal RNA (rRNA) genes [16, 23]. Although these data were derived from cell experiments, we thought PARP1 would promote the development of NT embryos by improve the two abnormal. X chromosome inactive starts from 72 hours after fertilization or activation when the embryos nearly develop to the blastocyst stage [24]. We have confirmed that Parp1 increased in CCNT blastocysts and the interference effect reached the strongest after 72 hours transfection when the embryos mostly develop to blastocysts. So we carried out the experiment under this condition. It is regrettable that NT embryos did not develop well as Parp1 was interfered, we thought it may be because of the role of PARP1 in DNA damage and repair [14]. And we guess the data of PARP1 in cell experiments maybe not suitable to embryos, for the signal path in embryonic development was complicated.

Second, the low developmental potential of female NT mouse embryos might be attributed abnormal Xi on the active X chromosome [5, 25]. When both X chromosomes are inactive, numerous X chromosome-linked genes are not sufficiently expressed. Any method for ameliorating this abnormal Xi, such as knocking out *Xist* or using siRNA to knock down *Xist*, would potentially lead to remarkable improvements in cloning efficiency. We chose a female donor to implement NT, but we did not observe improved development. This finding may be attributed to the differences between females and males. Matoba et al. reported that RNAi-mediated knockdown of *Xist* rescued the impaired development of male cloned mouse embryos, but this effect was not observed in females [6, 25]. *Xist* siRNA injection was found to be largely effective in repressing *Xist* overexpression in female cloned embryos but failed to rescue them, likely due to an inability to mimic consistent monoallelic *Xist* expression in these embryos [25]. Similar to Oikawa’s results, we did not achieve any improvement female NT embryonic development through knocking down *Parp1*, which instead exacerbated developmental problems.

Third, RNA sequence analysis revealed that the three types of NT-Parp1si embryos were not the same: NT-Parp1si-1 embryos were more similar to Fertilized embryos, whereas NT-Parp1si-2/3 embryos were more similar to NT embryos (Supplementary Figure 3). This finding indicates that the Papr1si-blastocysts may have differed from each other due to individual differences or because the influence of *Parp1* siRNA was not identical in each NT embryo. To obtain accurate and reliable data, the number of samples should be increased in future studies.

Finally, PARP1 plays important roles in DNA damage repair during nuclear reprogramming. DNA repair should be completed before epigenetic reprogramming via chromatin modification. Osada, Nozaki [14] reported that NT eggs formed PPN after activation with a significantly increased efficiency in *Parp1*-null embryos compared with control NT embryos, indicating that PARP1 is involved in the rebuilding of chromatin structure after NT by sustaining DNA repair. Moreover, most *Parp1*-null embryos ceased development at the four-cell stage, similar to our results indicating a significant reduction during the development of NT embryos from the four-cell stage to the morula stage when *Parp1* expression was knocked down to 40% by RNAi (Table 1).

### RNAi-mediated knockdown of Parp1 affects embryonic development-related genes

In the present study, reducing *Parp1* expression did not rescue the expression of genes that are important for embryonic development. Our data showed that *Sox17* was rapidly reduced following siRNA-mediated knock down of *Parp1* in NT embryos, whereas no difference with the PARP1 control was noted in Fertilized embryos (Supplementary Tables 2, 3, 4). SOX17 is first detected in a “salt-and-pepper” distribution within the inner cell mass (ICM) and is subsequently restricted to the nascent primitive endoderm (PE) epithelium [26–28]. SOX17 is coexpressed in cells with PE markers, consistent with an involvement in PE specification. Expression of SOX17 can reinforce PE identity in wave 2-generated cells. SOX17 inactivation in implantation-delayed blastocysts results in disruption of the organization of the PE and premature parietal endoderm differentiation. Premature detachment and migration of nascent parietal endoderm cells over the TE layer are observed [29]. In our study, *Sox17* levels were reduced in NT-Parp1si embryos during development, potentially explaining why knockdown of *Parp1* did not significantly improve NT embryonic development.

Furthermore, we evaluated total mRNA in single embryos and identified several DEGs among the three types of embryos. We also discovered genes that presented alterations in NT embryos, including genes that were rescued in PARP1si compared with IVF embryos. Most of these genes were X-linked genes, such as *Magea3*, *Magea6*, *Msn* and *Rhox6*. RHOX6 is regulated by the histone demethylase KDM6A [30]. KDM6A is an H3K27me3-specific histone demethylase that participates in Xi and has the opposite effect of the H3K27me3-specific histone methylase EZH2/EED. Most NT embryos fail to develop to term due to undefined reprogramming defects. H3K9me3 in the donor-cell genome is a major barrier to efficient reprogramming by NT. KDM4D removes H3K9me3 from the donor-cell genome and facilitates reprogramming. Matoba S et al. indicated that ectopic expression of the histone H3 lysine 9 trimethylation (H3K9me3) demethylase KDM4D greatly improves NT efficiency [1]. Both KDM6A and KDM4D belong to the KDM family; these proteins remove the methyl groups from H3. The inactivated X chromosome harbours both H3K27me3 and H3K9me3 marks; therefore, we hypothesize that overexpression of KDM6A might improve NT efficiency.

In the present study, we amplified RNA to rebuild the cDNA of single blastocysts. Through investigating gene expression in individual blastocysts, we hypothesized that we would identify more DEGs, but this was not proven to be the case. We used cDNA amplified from single blastocysts, where even a small change in a single blastocyst would be expected to be amplified into a thousand-fold change. Specific expression should be controlled under a limiting threshold or it will interfere with the formation of an accurate conclusion. To ensure stable and credible data, the number of samples should be increased in future studies.

In the present study, we used PARP1 as a factor to interfere with Xi because of the role that PARP1 plays in heterochromatin formation [7]. Our results showed that Xi was partially reversed in NT embryos after RNAi-mediated knock down of *Parp1*, but development was not improved. These findings indicate that PARP1 might be involved in Xi, but not through the *Xist* signalling pathway. Based on the significant improvement in NT efficiency achieved through overexpressing KDM4D, we believe that KDM6A up-regulation might improve NT efficiency.

## MATERIALS AND METHODS

### Ethics statements

This study was carried out in strict accordance with the regulations of the Instructive Notions with Respect to Caring for Laboratory Animals issued by the Ministry of Science and Technology of China. The animal experiment protocol was approved by the Harbin Medicine University Ethics Committees. All efforts were made to minimize suffering of the tested animals.

### Animals

B6D2F1 (C57BL/6 × DBA/2) female and male mice were obtained at 8 to 10 weeks of age from Vital River (Beijing, China).

### Cell culture

CCs (cumulus cells) were obtained during oocyte collection, washed twice in HEPES-buffered CZB medium (HEPES-CZB), resuspended in HEPES-CZB containing 3% PVP (polyvinylpyrrolidone, Sigma, PVP360 Saint Louis, MO) and directly used as donor cells (G0/G1) for NT. MEFs were isolated from 13.5 post-corium B6D2F1 mouse foetuses as previously reported [11]. Immature Sertoli cells from B6D2F1 males (3–7 days old) were used unless otherwise stated. Testes (2 per experiment) were placed in DPBS, and the tunica albuginea was removed. Masses of seminiferous tubules were incubated in DPBS containing 0.1 mg/mL collagenase (Sigma, C0130, Saint Louis, MO) for 30 min at 37°C. The tissue suspensions were then pipetted repeatedly with a 1- to 2-mm tip-diameter pipette to disrupt tubule fragments. The disrupted tubules were subsequently treated with 0.2 mg/mL trypsin (Sigma, T0303, Saint Louis, MO) for 5 min to loosen the cell aggregates, which were predominantly composed of Sertoli cells and germ cells. The final cell suspension for nuclear transfer was obtained after washing with DPBS and centrifugation. Immature Sertoli cells were identified as the smallest testicular cells (approximately 8 mm in diameter) [31]. MEFs were cultured in DMEM containing 10% FBS under 5% CO_2_ in humidified air at 37°C.

### Oocyte, fertilized zygotes recovery and spermatozoa preparation

Female B6D2F1 mice were superovulated via intraperitoneal injection of 5 IU pregnant mare serum gonadotropin (NSH, Ningbo, China) followed by 5 IU human chorionic gonadotropin 48 h later (NSH, Ningbo, China). Oocytes were collected from the oviducts 14 h after human chorionic gonadotropin injection. CCs were removed from the oocytes with 300 μg/mL hyaluronidase (Sigma, H4272, Saint Louis, MO) in HEPES-CZB by pipetting. Denuded oocytes with homogeneous ooplasm were selected and maintained in new droplets of CZB medium containing 5.6 mM glucose (CZBG), covered with sterile mineral oil (Sigma, M8410, Saint Louis, MO) and subsequently cultured at 37°C in a 5% CO_2_ atmosphere until use. *In vivo*-fertilized zygotes were collected 20 h post-human chorionic gonadotropin from the oviduct ampullae of superovulated C57BL6 females that had been mated with the DBA males. After removing cumulus cells with 300 μg/mL hyaluronidase in HEPES-CZB medium, zygotes were cultured in KSOM medium until use. Spermatozoa were collected from the cauda epididymis of 8- to 12-week-old B6D2F1 males, maintained in CZB-HEPES medium and prepared for injection.

### Generation of ICSI and NT embryos

ICSI was performed with a piezo-driven unit using methods described elsewhere except that our experiment was performed in HEPES-CZB containing 5 μg/mL cytochalasin B (Sigma, C6762, Saint Louis, MO) at room temperature. Only the sperm head was injected into the oocyte. After 30 min of recovery, the ICSI-generated embryos were washed several times and cultured in K-modified simplex optimized medium (KSOM) at 37°C in a 5% CO_2_ atmosphere. To eliminate any possible effects attributed to the NT methods from the following investigation, we adopted a one-step micromanipulation technique to reconstruct CC nuclear transfer (CCNT) and Sertoli cell nuclear transfer embryos, as described previously with modifications [32]. The outer diameter of the injection pipettes used for CC/Sertoli cell injection was 9 μm. Briefly, the donor cell membrane was disrupted with several Piezo pulses, and 4 to 7 cells were then sucked into the injection pipette. The oocyte MII spindle was adjusted to 8 to 10 o’clock, and one donor cell was injected into the nearby plasma. The spindle was immediately aspirated into the injection pipette and removed from the oocyte. One hour after NT, the reconstructed CCNT embryos were activated with 5 mM SrCl_2_ (Sigma, 439665, Saint Louis, MO) in Ca^2+^-free CZB containing 5 μg/mL CB for 6 h. Then, the embryos were washed in KSOM and cultured under the same conditions as the ICSI embryos.

### Preparation of siRNAs

Synthetic siRNA duplexes were designed by Stealth Designer (Life Technologies, Beijing, China), with the following sequences: *Parp1*, 5′-AAGCGUCGCUCUUAAAGACCAGCUG-3′ and 5′-CAGCUGGUCUUUAAGAGCGACGCUU-3′; negative control (Invitrogen Medium GC Duplex Cat. No.12935-3000, Carlsbad, CA). siRNA duplex mixtures were prepared as 100 nM stock solutions and stored at –80°C until use.

### Parp1si in cells and NT embryos

For experiments in cells, *Parp1* siRNA was transfected into MEF cells with RNAiMAX (Invitrogen, Cat. No.13778075, Carlsbad, CA) for 24 h. For NT embryos, *Parp1* siRNA (100 nM) was incubated with CCs for 1 h before reconstruction.

### Immunofluorescent detection of H3K27me3

Embryos were collected at 72 h post-activation (hpa) (i.e., after sperm injection). They were subsequently fixed in 4% (m/v) paraformaldehyde for 30 min and permeabilized with 1% (v/v) Triton X-100 for 50 min. After blocking with 1% (m/v) bovine serum albumin (Sigma, A9418, Saint Louis, MO) in PBS for 1 h, the embryos were incubated with an anti-H3K27me3 antibody (Millipore, Temecula, CA, 1:100) overnight at 4°C, followed by incubation with Alexa Fluor 488 mouse anti-rabbit IgG (Invitrogen, Carlsbad, CA, 1:100) for 1 h. After the nuclei were stained with 10 μg/mL Hoechst 33342, the embryos were mounted on slides with DABCO (1,4-diazabicyclo-(2.2.2) octane; Beyotime, P0126, Beijing, China) and observed with a laser-scanning confocal microscope (Zeiss, LSM700, Jena, Germany).

### Q-PCR evaluation of X chromosome-linked gene expression

Embryos were collected at blastocyst stages. RNA extraction and Q-PCR evaluation were performed as previously described with some modifications [33]. Total RNA was extracted from embryos using an RNeasy Mini kit (Qiagen, 74104) according to the manufacturer’s instructions. Total RNA was extracted from MEF cells with TRIzol reagent (Invitrogen, Carlsbad, CA) according to the manufacturer's protocol. cDNA was synthesized from total RNA using a High-Capacity cDNA Reverse Transcription kit (ABI, 4368814, Carlsbad, CA) in a total volume was 20 μL (2 μL of 10×RT buffer, 0.8 μL of 25× dNTP Mix, 1 μL of MultiScribe™ Reverse Transcriptase, 1 μL of oligo dT primer, 2 μL of 10× RT Random Primers, 1 μg of RNA, and RNase-free dH_2_O up to 20 μL). Q-PCR was performed on a CFX96 Realtime System (Bio-Rad) using a 1 μL of synthesized cDNA, 10 μL of TransStart™ Top Green Q-PCR SuperMix (TransGen, AQ131, Beijing, China), and gene-specific primers in a 20-μL reaction volume. The thermal cycling conditions were initial denaturation at 95°C for 3 min and 42 cycles comprising the following steps: 10 s at 95°C for DNA denaturation, 10 s at 60°C for primer annealing and 30 s at 72°C for primer extension. The melting protocol ranged from 65°C to 95°C (increment: 0.5°C/5s). The threshold cycle (Ct) value represents the cycle number at which the fluorescence of the sample is significantly higher than the background. Reactions were conducted according to the protocol provided with the Haigene SYBR green quantitative PCR kit. The PCR products were analysed by generating a melting curve. The relative amount of gene expression was analysed via the 2-ddCt method, which is a convenient method for analysing relative changes in gene expression in real-time quantitative PCR experiments. The amplification specificity of each Q-PCR assay was confirmed by performing a melting curve analysis to verify that the primers used could amplify only one specific PCR product. The amplification efficiencies were calculated according to the following formula: efficiency (%)= (3^(-1/slope)-1^)×100. The amplification efficiencies of all the tested genes ranged from 94% to 110%, and all correlation coefficients were >0.99. These results demonstrated that the synthesized primer sequences were accurate and suitable for the experiments (Table 2). The assay included a no-template control (NTC), which indicated background activity. All Q-PCR reactions were biologically and technically conducted in triplicate, and ten blastocysts from each group composed a single replicate. The specificity of the Q-PCR reaction was confirmed using single peaks in the melt curves. *H2afz* and *Cyca* were employed as candidate references for embryos, and *Gapdh* was used for cells.

**Table 2:** Primers used for the detection of rDNA and X chromosome-linked gene expression in cells and embryos

Gene	Gene no.		Primer sequence (5’-3’)	E	R^2^
47S rRNA	V00850	Sense Antisense	CTCCTGTCTGTGGTGTCCAA GCTGGCAGAACGAGAAGAAC	93.0	1.000
18S rRNA	BK000964	Sense Antisense	CGCGGTTCTATTTTGTTGGT AGTCGGCATCGTTTATGGTC	101.3	0.999
Parp1	NM_007415.2	Sense Antisense	GGCAGCCTGATGTTGAGGT GCGTACTCCGCTAAAAAGTCAC	109.5	0.997
Xist	NC_000086.7	Sense Antisense	AGTGCTCTATACGTGGCGGT ATGCAACCCCAGCAATAGTC	106.9	0.997
Magea3	NP_064401	Sense Antisense	CAGAGCCTACCCTGAAAAGTATG AGCATCTGTTCAAGATCCAGGT	96.5	0.996
Magea6	NP_064403	Sense Antisense	CCCAAGGGCTCTTGCAGAAA AATGGTCAGAGAAATTGGAGCAT	98.0	0.997
Msn	NM_010833	Sense Antisense	TCTTATGCCGTCCAGTCTAAGT GGTCCTTGTTGAGTTTGTGCT	94.8	0.994
H2afy (macroH2A1)	NC_000079.6	Sense Antisense	CGGTGGTGAAGTAGGAAACAC GCTGCCAATGGATGGGAAG	96.2	0.998
Cyclophilin-A	NM_008907	Sense Antisense	GAGCTCTGAGCACTGGAGAGA CCACCCTGGCACATGAAT	96.0	0.998
H2afz	NM_016750.2	Sense Antisense	TTCCCGATCAGCGATTTGTGGA ACAGCGCAGCCATCCTGGAGTA	95.6	0.999
Gapdh	NM_008084	Sense Antisense	AAACCTGCCAAGTATGATGA GTGGTCCAGGGTTTCTTACT	88.9	0.999

E indicates amplification efficiency. R^2^ indicates regression coefficients.

### Illumina sequencing, De Novo assembly and functional annotation

For Illumina sequencing, total RNA was isolated from single blastocysts. After poly(A) mRNA was purified and fragmented into small pieces, we used random hexamer primers and reverse transcriptase (Invitrogen, Carlsbad, CA) to perform first-strand cDNA synthesis. Second-strand cDNA synthesis was conducted with RNase H (Invitrogen, Carlsbad, CA) and DNA polymerase I (New England BioLabs, Ipswich, MA). We constructed a cDNA library with average insert sizes of 200 to 500 bp and conducted cDNA sequencing using the Illumina HiSeq™ 2000 system according to the manufacturer’s protocols, with a read length of 100 bp. The average proportion of clean reads for the library was 99.73%. We screened differentially expressed genes (DEGs) based on analysis of the Poisson distribution [34] with an FDR<0.05. All the sequencing and analysis work was completed by the HuaDa Gene Company.

### Experimental design and statistical analysis

Each experiment was repeated at least three times. All the embryos were randomly allocated to each treatment group. Blastocyst formation rate was analysed using the χ^2^ test. More than five embryos were selected from each group for observations of H3K27me3 using a confocal microscope. In the Q-PCR experiments, ten blastocysts from each group were used as a single replicate. Statistical comparisons were analysed using one-way ANOVA. The relative abundances of gene transcripts were established by testing the data for normality and equal variance using the Levene median test and ANOVA and were followed by multiple pair wise comparisons using Tukey’s test. Differences of P<0.05 were considered statistically significant. The differences in mRNA expression were analysed using SPSS 19.0.

## SUPPLEMENTARY MATERIALS FIGURES AND TABLES









## Abbreviations

PARP: Poly (ADP-ribose) polymerase
Xi: X chromosome inactivation
NT: nuclear transfer
ICSI: intracytoplasmic sperm injection
*Xist*: X-inactivation-specific transcript
rDNA: ribosomal DNA
CCs: cumulus cells
CCNT: CC nuclear transfer
