# Supplementary material for: RNAi-mediated knockdown of Parp1 does not improve the development of female cloned mouse embryos
Source: Oncotarget. 2017 Jul 18;8(41):69863–73. doi: 10.18632/oncotarget.19418 (PMC5642522; doi:10.18632/oncotarget.19418)
Supplement: Supplementary file 1 [file oncotarget-08-69863-s001.pdf]

RNAi-mediated knockdown of *Parp1* does not improve the development of female cloned mouse embryos

SUPPLEMENTARY MATERIALS

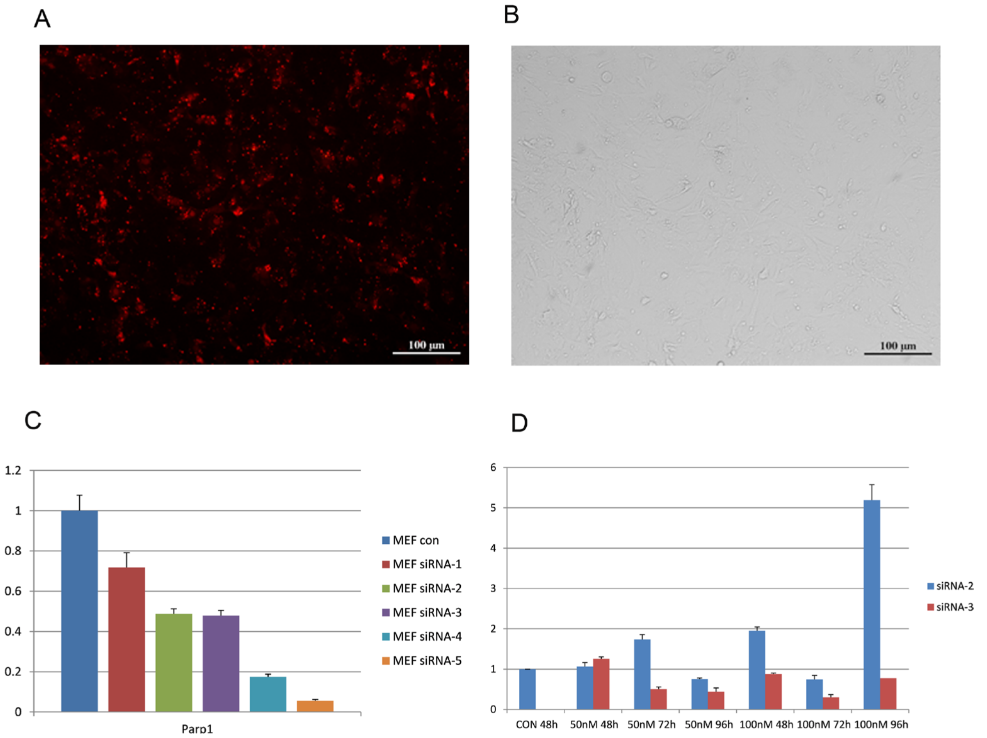

Supplementary Figure 1: Detection of *Parp1* siRNA efficiency. (A) MEFs were transfected with fluorescent control RNA. **(B)** Bright light control. **(C)** MEFs were transfected with siRNA-1, siRNA-2, siRNA-3, siRNA-4, or siRNA-5 at 100 nM for 72 h, and *Parp1* expression was detected via Q-PCR. **(D)** MEFs were transfected with siRNA-2 or siRNA-3 at 50 or 100 nM for 48, 72, or 96 h, and *Parp1* expression was detected via Q-PCR.

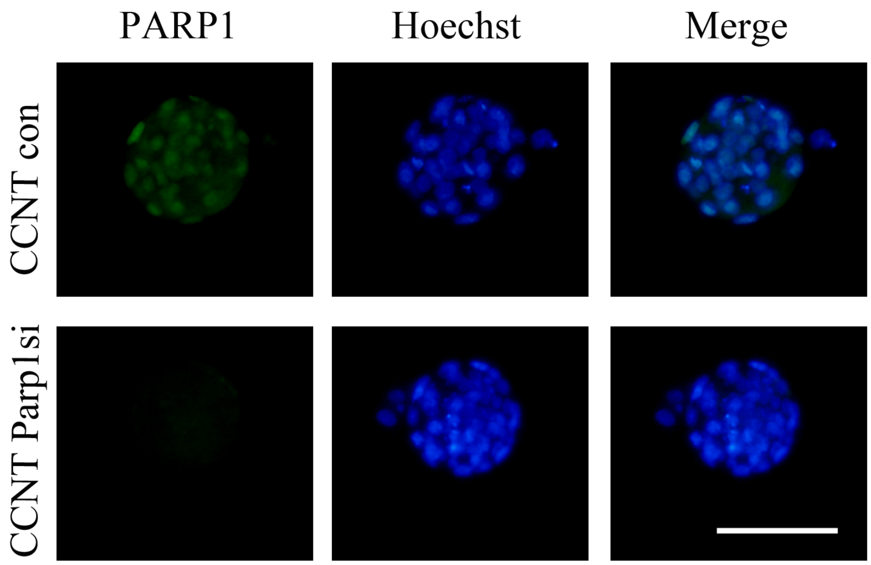

Supplementary Figure 2: Immunofluorescent detection of PARP1 in CCNT-Parp1si and CCNT blastocysts.

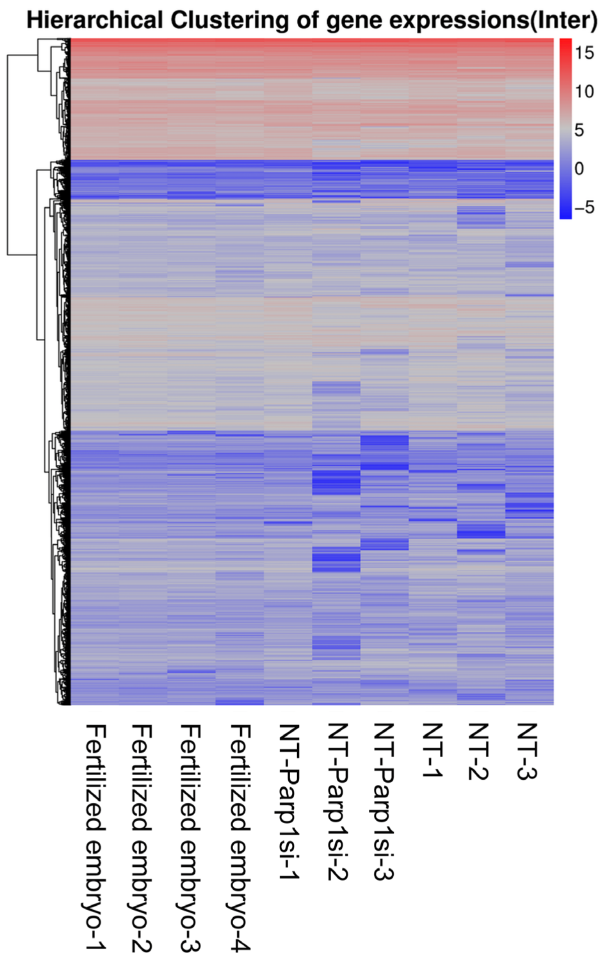

Supplementary Figure 3: Heatmap illustration of all genes expressed in the three types of blastocysts. Clustering of embryos.

Supplementary Table 1: Sequence of mouse Parp1 siRNA

NO.	Initiation site	Sequence	Score
1	524	CCGCTGGTACCATCCAACTTGCTTT	52.0
2	2602	ACAAGCCCTTCAGGCAGCTTCACAA	52.0
3	1856	CAAACTTGAGCAGATGCCCTCCAAA	48.0
4	2719	CAGGCTACATGTTTGGGAAAGGGAT	48.0
5	963	CAGCTGGTCTTTAAGAGCGACGCTT	52.0

Supplementary Table 2: Differential expressing genes between IVF and NT blastocysts

See Supplementary File 1

Supplementary Table 3: Differential expressing genes between NT and NT-Parp1si blastocysts

See Supplementary File 2

Supplementary Table 4: Differential expressing genes between IVF and NT-Parp1si blastocysts

See Supplementary File 3
